# Supplementary material for: The causal role of circulating inflammatory markers in osteoporosis: a bidirectional Mendelian randomized study
Source: Front Immunol. 2024 Jul 18;15:1412298. doi: 10.3389/fimmu.2024.1412298 (PMC11291241; doi:10.3389/fimmu.2024.1412298)
Supplement: Supplementary file 1 [file DataSheet_1.zip › supplementary material/CIM-OSTEOPOROSIS /GCST90274844.csv_leave_one_out.pdf]

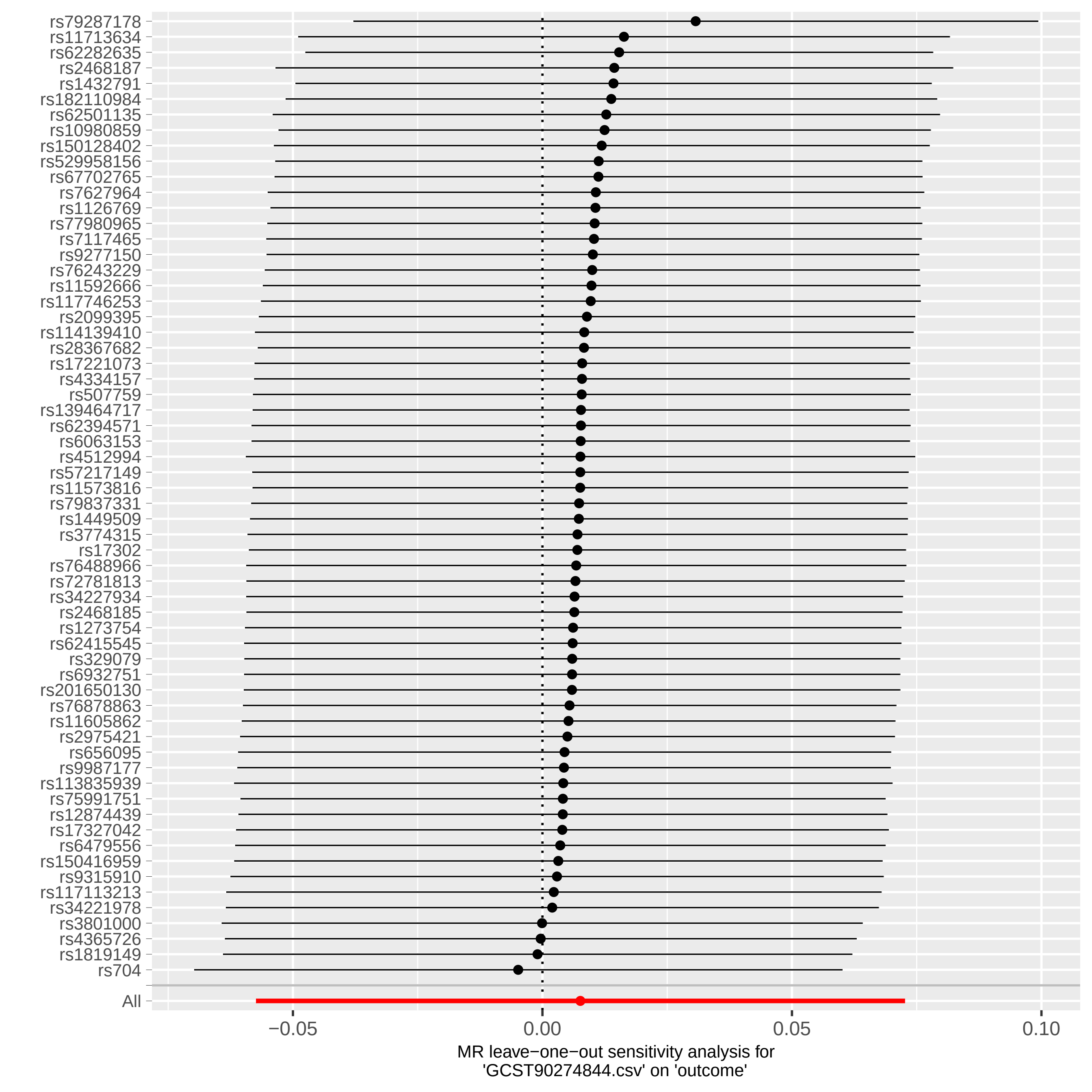
rs79287178
rs11713634
rs62282635
rs2468187
rs1432791
rs182110984
rs62501135
rs10980859
rs150128402
rs529958156
rs67702765
rs7627964
rs1126769
rs77980965
rs7117465
rs9277150
rs76243229
rs11592666
rs117746253
rs2099395
rs114139410
rs28367682
rs17221073
rs4334157
rs507759
rs139464717
rs62394571
rs6063153
rs4512994
rs57217149
rs11573816
rs79837331
rs1449509
rs3774315
rs17302
rs76488966
rs72781813
rs34227934
rs2468185
rs1273754
rs62415545
rs329079
rs6932751
rs201650130
rs76878863
rs11605862
rs2975421
rs656095
rs9987177
rs113835939
rs75991751
rs12874439
rs17327042
rs6479556
rs150416959
rs9315910
rs117113213
rs34221978
rs3801000
rs4365726
rs1819149
rs704
All
−0.05
0.00
0.05
0.10
MR leave−one−out sensitivity analysis for
'GCST90274844.csv' on 'outcome'
